# Circulating tumour cells in the -omics era: how far are we from achieving the ‘singularity’?

**DOI:** 10.1038/s41416-022-01768-9

**Published:** 2022-03-10

**Authors:** Tanvi H. Visal, Petra den Hollander, Massimo Cristofanilli, Sendurai A. Mani

**Affiliations:** 1 Department of Translational Molecular Pathology, The University of Texas MD Anderson Cancer Center, Houston, TX USA; 2 Department of Medicine-Hematology and Oncology, Robert H. Lurie Comprehensive Cancer Center, Feinberg School of Medicine, Northwestern University, Chicago, IL USA

**Keywords:** Cancer, Cell biology

## Abstract

Over the past decade, cancer diagnosis has expanded to include liquid biopsies in addition to tissue biopsies. Liquid biopsies can result in earlier and more accurate diagnosis and more effective monitoring of disease progression than tissue biopsies as samples can be collected frequently. Because of these advantages, liquid biopsies are now used extensively in clinical care. Liquid biopsy samples are analysed for circulating tumour cells (CTCs), cell-free DNA, RNA, proteins and exosomes. CTCs originate from the tumour, play crucial roles in metastasis and carry information on tumour heterogeneity. Multiple single-cell omics approaches allow the characterisation of the molecular makeup of CTCs. It has become evident that CTCs are robust biomarkers for predicting therapy response, clinical development of metastasis and disease progression. This review describes CTC biology, molecular heterogeneity within CTCs and the involvement of EMT in CTC dynamics. In addition, we describe the single-cell multi-omics technologies that have provided insights into the molecular features within therapy-resistant and metastasis-prone CTC populations. Functional studies coupled with integrated multi-omics analyses have the potential to identify therapies that can intervene the functions of CTCs.

## Introduction

Liquid biopsies are minimally invasive and have the potential to provide diagnostic and prognostic information that can aid in the treatment of patients with various solid tumours [1–3]. Circulating tumour cells (CTCs) and circulating tumour DNA (ctDNA), two prime biomarker candidates detected through liquid biopsy, can provide insight into tumour evolution, tumour biology, cancer progression and therapy resistance [4–6]. CTCs and ctDNA have great translational potential, and a plethora of clinical trials are currently utilising CTCs and ctDNA as biomarkers. These markers reflect different biological aspects of the disease, with CTCs crucial for evaluating prognosis in early and advanced disease (colorectal cancer, breast cancer and prostate cancer) [7] and ctDNA vital for monitoring treatment response and relapse [8, 9]. Despite active research in the ctDNA field, the lack of standardised methods for ctDNA extraction and analysis makes its utility controversial.

Research over the past decade has resulted in immense improvements in methods for CTC detection, separation, microfluidic isolation and genomic analysis. Whereas ctDNA can only be analysed at a genomic level, CTCs can be dissected at transcriptomic, genomic and proteomic levels either in bulk or as single cells. The precision medicine era has led to an in-depth investigation of CTCs. In this review, we discuss what is known about CTC biology and explore how single-cell multi-omics has contributed to our current understanding of these rare cancer cells and their potential in personalised medicine.

CTCs are shed from the primary tumour and are directly involved in the metastatic cascade based on Paget’s “seed and soil” hypothesis. CTCs are extraordinary cancer cells disseminated from the primary tumour that survive in the systemic circulation and initiate tumour formation at a site distant from the primary tumour. During the metastatic process, cancer cells undergo the epithelial-mesenchymal transition (EMT), through which they acquire the migratory and invasive ability and invade the surrounding stroma. They then intravasate and survive in the vasculature as CTCs, eventually extravasating at distant organs resulting in metastatic tumours [10, 11]. Although this cascade is very complex, it is inefficient as only a small fraction of cells progresses through all these stages. Not every CTC in the vascular system, and even fewer have the ability to establish metastases [12, 13].

The mesenchymal-epithelial transition (MET), which is the reverse of EMT, plays a crucial role in the colonisation and formation of macro-metastases. CTCs can exist in different states across the EMT spectrum [14, 15]. However, little is known regarding the influence of EMT and MET on CTC biology. CTCs can migrate as single cells or as clusters [16], and both single cells and clusters have been shown to have metastatic and invasive potential [17, 18]. CTCs are heterogeneous both within an individual patient and across cohorts. Properties, including cell size, cell-surface markers and tumour-initiating potentials, differ [19, 20]. In addition, accumulating evidence suggests that components of the tumour microenvironment bolster the metastatic potential of CTCs [21–25].

CTCs serve as a privileged gateway to study mechanisms of metastasis and have prognostic and predictive value in breast and other cancers [26–31]. The ability to non-invasively draw blood frequently allows for longitudinal sampling, and thus liquid biopsy is a better strategic approach for a personalised and precise treatment strategy for cancer patients than is tissue biopsy [32, 33]. Advancements in single-cell technologies have allowed the identification of rare subpopulations of CTCs and the study of their dynamics. This review summarises relevant clinical and biological properties of CTCs, technical aspects of CTC analysis, the use of CTC analysis as a powerful tool in clinical diagnosis and prognosis and the potential for CTC-targeting therapies.

## CTC heterogeneity—through the EMT lens

The activation of EMT occurs at the early stage of metastatic progression. During EMT, carcinoma cells lose their ability to adhere to other cells and the matrix, lose apicobasal polarity and gain mesenchymal characteristics. The gain of mesenchymal-like features enables cells to migrate from the primary tumour [34] and promotes their entry into the circulation [35, 36]. During EMT, expression of epithelial markers such as E-cadherin, EpCAM and cytokeratins is lost and there is a gain in mesenchymal markers such as N-cadherin, vimentin and fibronectin [37, 38]. EMT is a reversible process that is driven by tumour-intrinsic as well as tumour-extrinsic mechanisms mediated by growth factors, including transforming growth factor beta (TGF-β) and epidermal growth factor (EGF), by EMT-inducing transcription factors such as Snail, Twist, Slug, ZEB1 and FOXC2, and by the suppression of microRNAs (miRNAs) such as miR-200 and miR155 [39–45]. The EMT program enhances tumorigenicity, metastatic ability, radio-resistance and chemoresistance [46–50]. The EMT program also imparts stem cell-like properties, including self-renewal ability, to cancer cells [51–53].

**Fig. 1:**
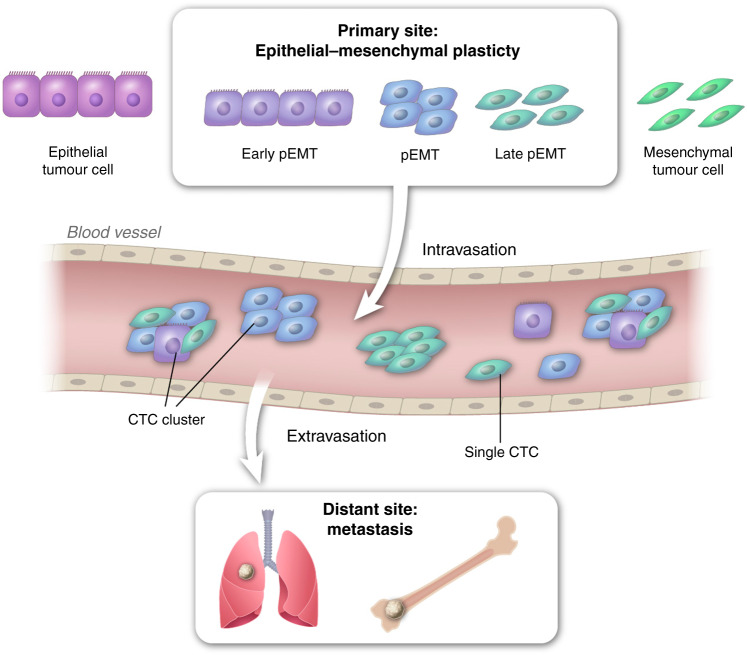
Impact of CTC E/M plasticity on metastasis. A small proportion of carcinoma cells exhibit E/M plasticity and display an increased capacity for intravasation and survival in the vasculature. Such CTC clusters extravasate and form metastatic lesions.

EMT is a spectrum rather than a process with binary states [54–56]. Three types of CTCs have been described: epithelial-like, mesenchymal-like and hybrid epithelial/mesenchymal (E/M) phenotypes [57]. CTCs that display a hybrid E/M phenotype have the plasticity to interconvert between epithelial and mesenchymal states enabling adaptation to the changing tumour microenvironment (Fig. 1) [58]. Invasion of the basement membrane by tumour cells is essential for metastatic cascade [40]. Following intravasation, stem cell-like features of CTCs help resist fluid shear stress [59–61]. To establish a metastasis, CTCs have to regain their epithelial phenotype [62]. Thus, CTCs undergo EMT when they start their journey from the primary tumour, and MET to form a metastatic lesion (Fig. 1) [63–65]. Cellular plasticity is linked with tumour progression, enhanced migration, metastasis and therapy resistance [66–70] and CTCs with E/M hybrid phenotypes and stem cell-like signatures are correlated with significantly reduced progression-free survival [71] and more aggressive and metastatic disease [72, 73]. CTC clusters with E/M hybrid plasticity display stemness, indicating that EMT contributes to the self-renewal of CTCs [74].

The detection and isolation of CTCs from the blood is routinely performed using the epithelial marker EpCAM [75], but levels of this marker are significantly reduced or completely lost on CTCs that have undergone EMT. In breast cancer patients, it has been reported that CTCs are enriched in mesenchymal markers (N-cadherin, TWIST and Vimentin) and have decreased epithelial marker expression (E-cadherin, EpCAM and CK8/18/19) [14]. In a study involving patients with progressive colon cancer, CTCs expressed transcription factors that initiate EMT (FOXC2, SNAIL and TWIST1) and vimentin but have low levels of epithelial markers E-cadherin and EpCAM [76]. Lecharpentier et al. demonstrated that single CTCs as well as clusters of CTCs from patients with non-small-cell lung cancer co-expressed the mesenchymal marker vimentin and the epithelial marker keratin [64]. These findings underscore the need for the discovery of markers that can capture CTCs independently of EMT state. Vimentin might be a suitable candidate [77].

CTCs with hybrid E/M properties express aldehyde dehydrogenase 1 (ALDH1), which is associated with stemness, and is expressed in hybrid E/M CTCs together with cytokeratins and nuclear TWIST1 [78] these CTCs are chemo-resistant [79]. CTCs isolated from patients with endometrial cancer display E/M plasticity and stemness resulting in enhanced survival and chemoresistance [80]. Collectively, these studies suggest that hybrid E/M CTCs are more metastatic than epithelial-like or mesenchymal-like CTCs and targeting hybrid E/M CTCs could improve patient survival. However, additional phenotypic and molecular characterisation of hybrid E/M CTCs is necessary.

## CTC capture techniques

CTC detection could play an important role in the early detection of cancer and recurrence. Phenotype identification and molecular analysis of CTCs are expected to yield a significant amount of information about metastatic disease progression. However, CTCs are rare cells in the blood, are present at very low and undetectable levels. This poses a major challenge for detection, isolation and further downstream applications. Due to their rarity, a preceding CTC enrichment step is essential to separate CTCs based on the physical and biological properties of the tumour cells.

Several CTC detection platforms have emerged during the past decade. These platforms exploit a specific CTC feature and/or function for isolation, enumeration and functional characterisation. CellSearch (Janssen Diagnostics) was the first FDA-approved technology for CTC capture. It is based on the selection of EpCAM^+^ CTCs [81]. The Adna Test (Adnagen) uses captures CTCs with antibody-coated magnetic beads and has been shown to detect CTCs in blood of prostate and metastatic breast cancer patients [82, 83]. The MACS system employs a cytokeratin-based CTC counting approach [84], and geometrically enhanced differential immunocapture (GEDI), which has high specificity, captures CTCs on microfluidics with immobilised antibodies to proteins such as HER2 and PSMA [85]. Other microfluidics-based technologies utilise dual-modality approaches to capture CTCs using flow and immunomagnetic beads (IsoFlux) [86], viscous flow stress and magnetic force (the Quadrupole Magnetic Separator) [87], or a combination of microfluidics with sequential positive and negative CTC enrichment on a microchip (CTC-iChip) [88]. Such technologies enable robust molecular characterisation of rare cells like CTCs and have the potential to identify patients with nascent-stage disease.

Apart from label-dependent techniques, CTCs can be isolated using a plethora of marker-independent approaches based on physical properties, such as size [89, 90], charge [91, 92], density [93, 94] and elasticity [95, 96]. Moreover, CTCs can be isolated based on their biological properties, such as their invasive capability [97] or the presence of specific surface antigens that distinguish CTCs from other immune cells in the blood (negative selection of immune cells to enrich CTCs) [88, 98]. Isolation by size of tumour cells (ISET) is a filter-based isolation and enrichment technique that has higher efficiency than CellSearch [99, 100]. Similar approaches include the MetaCell system [101], ScreenCell Cyto [102] and CellSieve [103]. Although readily automated, filter-based exclusion is often prone to pore-clogging and requires large amounts of blood.

Additionally, the viability of CTCs in blood and purity of blood samples are major challenges [104]. The Cyttel system combines anti-CD45 immunohistochemistry and fluorescence in situ hybridisation (FISH) and involves the application of density-based centrifugation; it has a high detection rate in non-small cell lung cancer samples [105]. Other platforms, including OncoQuick [106], Percoll and Ficoll-Hypaque [107], are used to segregate whole blood components for CTC isolation; however, the formation of CTC aggregates interferes with these methods. Direct imaging offers an advantage over size-dependent isolation as it enables CTC detection and localisation using markers, such as cytokeratins, DAPI and CD45 and excludes the need for enrichment. FASTcell, CytoTrack and ImageStream are some of the platforms used in clinical studies to detect individual CTCs and clusters of CTCs as biomarkers [108–110]. Overcoming limitations of low sensitivity and resolution will make these powerful platforms for CTC detection and enumeration.

## The CTC armour

**Fig. 2:**
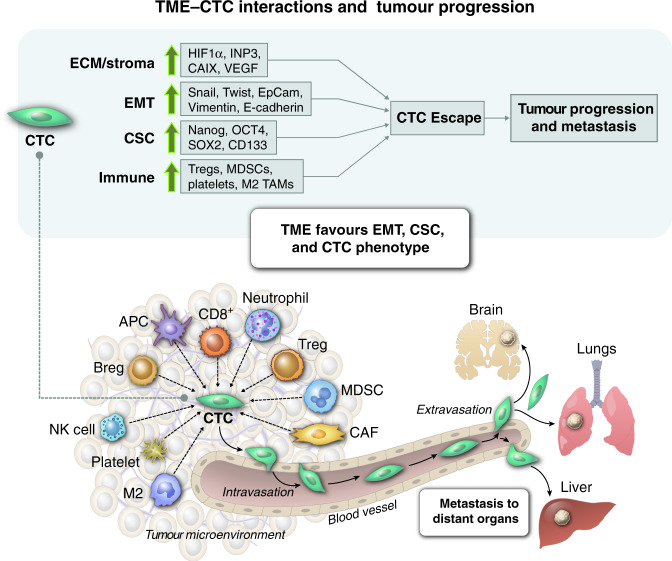
The impact of tumour cell-intrinsic and extrinsic factors on establishing CTCs with E/M plasticity. Upregulation of genes related to hypoxia (*HIF-1a*, *INP3*, *CAIX* and *VEGF*), EMT *(SNAIL*, *TWIST*, *EpCAM*, *Vimentin* and *E-cadherin*), cancer stem cells (*Nanog*, *OCT4*, *SOX2* and *CD133*) and immune cell function in the tumour microenvironment remodels CTCs and primes these cells for escape from primary tumour site.

CTCs shield themselves with protective armour to withstand the pressures of the microenvironment. Survival of CTCs is influenced by hypoxia, autophagy, inflammation and immune suppression, which all favour cancer progression (Fig. 2). CTCs interact with various components in the blood that nurture their survival, and they receive cues from the blood microenvironment that contribute to resistance to immune effector cells, anoikis and chemotherapy [111, 112]. CTCs express various cancer stem cell markers associated with decreased sensitivity to chemotherapies and cytotoxic immune effector cells [22, 113]. Further, high-resolution copy-number analysis revealed genetic alterations in CTCs in genes related to cancer cell dormancy, such as *AKT2*, *PTEN* and *CADM2*, genes associated with invasion and metastasis, such as *ANGPTL4*, *BSG*, *miR-373* and *LTBP4* and anti-apoptosis genes, such as *miR-24*, *LTBP4*, *TFF3*, *NUMBL* and *miR-181* [114]. This suggests that CTCs can exist in subpopulations with different metastatic potentials.

Additionally, *survivin* mRNA was detected in peripheral blood of patients with breast cancer, metastatic colorectal cancer, esophageal squamous cell carcinoma and non-small-cell lung cancer [115–118]. Moreover, CTCs with high survivin expression is correlated with poor overall survival in patients with metastatic colorectal cancer [117]. Survivin inhibits apoptosis of cancer cells and assists in immune evasion via blockade of cytotoxic anti-tumour activity of natural killer (NK) cells [119]. Extracellular survivin has been shown to decrease NKG2D receptor levels and affect the functionality of NK cells through alteration of activation markers, such as perforin, granzyme B, TNF-α and IFN-γ [120]. HER2 also plays a role in cell survival and proliferation in CTCs by activating the PI3K/Akt and Ras/Raf/MAPK signaling pathways [121, 122].

The presence of upregulated immune-suppressive elements in the tumour microenvironment may also facilitate CTC survival. A study of hormone receptor-positive breast cancer patients showed that the immune checkpoint regulator PD-L1 is upregulated on CTCs [123]. PD-L1 mediates the activity of regulatory T cells, which exert immunosuppressive action and induce apoptosis of activated cytotoxic T cells [124]. CTCs can induce apoptosis of peripheral T-helper cells by upregulation of CD95L/FasL on their surface. Sustained activation of Toll-like receptor signaling may also allow CTCs to evade the immune system. This activation generally attracts host immune cells but can also escalate inflammation and have deleterious effects [125]. Cytokines like IL-6 and IL-8, which are secreted by osteosarcoma cells, play a role in recruitment and clonal expansion of CTCs in distant organs to promote osteosarcoma outgrowth and act as tumour-derived attractants of CTCs; this phenomenon is called ‘tumour self-seeding of CTCs’ and can accelerate tumour growth [126, 127]. Thus, CTCs are efficient at modulating the immune microenvironment and favor self-survival in the circulation.

Platelets also play essential roles in the survival of CTCs by rendering them resistant to shear stress and NK cell cytotoxicity [128, 129]. Proteins like P-selectin and integrins expressed on the platelet cell surface contribute to tumorigenesis by promoting adhesion between CTCs and platelets [130, 131]. Several mechanisms mediated by platelet-derived TGF-β enable the escape of CTCs from immune clearance. TGF-β-mediated downregulation of anti-apoptotic Bcl-2 influences apoptosis, differentiation and cell proliferation [132] and downregulates NKG2D expression, thereby impairing NK cell cytotoxicity [133]. Platelet aggregation serves as a shield for CTCs, as CTC-platelet complexes escape immune attacks [134]. Blockade of CTC-platelet interactions by modifying platelets to express TRAIL resulted in the eradication of tumour cells in a model of prostate cancer metastasis [135].

In summary, CTCs expressing survivin, HER2 or PD-L1 or with mutations mentioned above could be valuable indicators of the survival capacity of the CTCs and could be used for the prediction of recurrences and metastasis. Further, abrogation of CTC survival mechanisms could create an environment hostile for CTCs and reduce their potential for survival and metastasis, especially in breast and gastric cancers where HER2 is overexpressed. A better understanding of CTC heterogeneity and the phenotypic and genotypic characterisation of CTCs could identify the ‘culprit’ CTC populations responsible for cancer progression, which could be targeted therapeutically. Moreover, immunosuppression-dependent CTC survival mechanisms could be targeted to abrogate the metastatic cascade.

## Dissecting CTCs using a multi-omics microscope

### Singularity matters

**Fig. 3:**
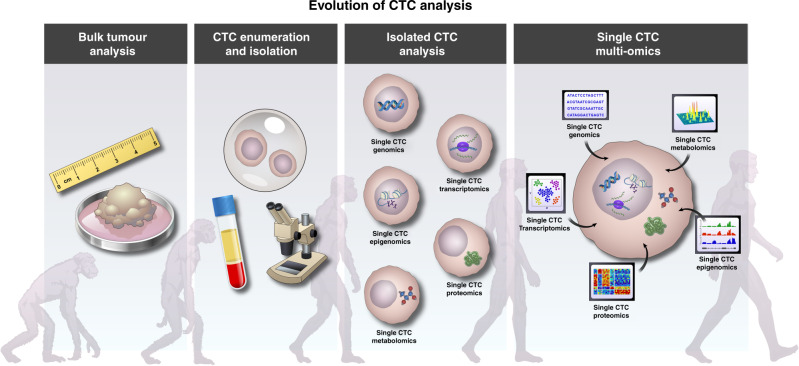
Evolution of CTC analysis. Development of sophisticated single-cell analysis technologies has allowed the dissection of heterogeneity within CTCs. Integration of multi-omics technologies will provide comprehensive knowledge of the landscape of CTC biology.

With the advent of various CTC capture technologies, research in the CTC field was no longer limited to detection and enumeration. Specifically, genomes, transcriptomes and proteomes of CTCs have been assessed. Single-cell multi-omics methods can dissect intra- and inter-tumour heterogeneity and provide insight into the roles of rare cells, such as CTCs, cancer stem cells and cells enriched for EMT [25, 136–140]. One single CTC can now be analysed to generate proteomic, transcriptomic and epigenomic data, yielding valuable information on molecular mechanisms and transcriptional programs active in these cells [141]. Since the analysis of CTCs gives insight into primary tumour characteristics, a minimally invasive yet maximally informative CTC multi-omics analysis would be an excellent approach to relate acquired genomic variation with changes in cellular function and phenotype in patients with different stages of cancer progression (Fig. 3). Therefore, CTC analysis should facilitate the personalisation of therapeutic options.

### Single-CTC genomics

Understanding the genome at single-cell resolution across various human tissues has changed the way we look at human anatomy and has led to the discovery of new cell phenotypes. The genomic analysis of CTCs has similarly increased our understanding of tumour metastasis, intra-tumour heterogeneity and genetic mutations [142–145]. Whole-genome amplification (WGA), performed using methods such as multiple displacement amplification (MDA), degenerate oligonucleotide-primed polymerase chain reaction, multiple annealing and looping based amplification cycles (MALBAC) or linear amplification via transposon insertion (LIANTI), yields accurate genomic analysis of CTCs if performed with precision [146, 147]. MDA-based methods are well suited for amplifying point mutations because of the higher fidelity than PCR-based methods. MDA has been used to analyse the genomes of patient-derived CTCs [143, 148]. MALBAC combines MDA- and PCR-based methods and generates, using a specific oligonucleotide design, looped DNA molecules during multiple initial rounds of displacement preamplification. These looped DNAs are then further amplified using PCR [149]. MALBAC has been successfully used to analyse single-nucleotide variant (SNVs) profiles in CTCs [150]. LIANTI amplifies T7 promoter-tagged DNA fragments into thousands of RNA copies; this method covers 97% of the genome with a reduced false-negative rate compared to other WGA methods and could be applied to single-CTC profiling [151].

Lu et al. systematically evaluated four different WGA methods for copy-number variation (CNV) profiling of single CTCs from non-small cell lung cancer patients. The group showed that MALBAC and MDA-based Repli-g methods yield significantly higher genomic coverage than PCR-based methods (GenomPlex and Ampli1) [152]. Another study by Malihi and colleagues used the Epic Sciences CTC platform for genomic characterisation of CTCs based on WGA analysis of copy-number alterations (CNA) to evaluated associations between single-CTC genomics and clinical features, progression-free survival and overall survival in prostate cancer [153]. It currently remains challenging to achieve high genome coverage, low allele dropout and low amplification errors; however, single-CTC genomic analysis has the potential to become a powerful non-invasive diagnostic tool to study gene expression changes in cancer patients with localised, metastatic and recurrent diseases.

### Single-CTC epigenomics

The cancer cell epigenome harbors myriad abnormalities that are potential cancer biomarkers and that could be targeted using therapeutic strategies [154]. Epigenetic modifiers are central components in regulating gene expression, and it is of prime importance to study epigenomes on the single-cell level to identify rare populations and characterise intra-tumour heterogeneity. During the past 20 years, mechanisms of epigenetic regulation have been thoroughly mapped and studied, and, with the emergence of single-cell technologies over the last 6 years, it has become possible to identify aberrations in major mechanisms like DNA methylation, chromosome conformation, histone modifications on a single-cell level [155–157].

Genes encoding tumour suppressors are silenced in CTCs of breast cancer patients [158, 159]; DNA methylation patterns and miRNA expression are altered in these CTCs [159–161]. DNA methylation and miRNA expression patterns provide crucial insights into molecular mechanisms of EMT and metastasis with important therapeutic implications [162]. Whole-Genome Bisulfite Sequencing (WGBS) offers a comprehensive DNA methylation profile in a broad range of biological systems. It can be used to analyse single-cell methylomes, whereas ChIP-seq allows the study of histone modifications on the single-cell level [163, 164].

DNA methylation signatures have been studied in CTCs of breast and prostate cancers [159, 165]. A CTC line was established using a mouse hepatocellular carcinoma model and overexpression of HGF and c-MET was linked with decreased DNA methylation at the promoters of genes that contributed to EMT [166]. Hypomethylation of stem cell genes and increased expression of pluripotency networks were also detected, suggesting increased metastatic potential due to the acquisition of a stem cell-like phenotype [18]. In prostate CTCs, increased methylation of promoters of known EMT-repressors (*miR-200c/141*, *miR-200b/a/429* and *CDH1*) and epigenetic regulation of EMT-associated genes during bloodborne dissemination were detected [167]. Thus, analysis of the methylation status of CTCs can inform about hybrid E/M states associated with malignant phenotypes.

miRNA dysregulation is associated with different pathologies including cancer [168]. miR-21 expression was elevated in keratin-positive CTCs in 11 out of 25 patients with metastatic breast cancer, as shown by single-CTC analyses using in situ hybridisation [169]. In these breast cancer patients, miR-21 is a marker of CTCs with an E/M plasticity phenotype as demonstrated by comparing miR-21 expression in CTCs that co-expressed miR-21 and keratin and by analysis of MCF-7 cells induced to undergo EMT that had lost keratin expression but retained miR-21. Another study showed elevated miR-10b expression in CTCs isolated from metastatic breast, colorectal and prostate cancer patients [170]. miR-10b is linked with tumorigenesis and metastasis and is a promising target for miRNA-based therapy [171]. Analysis for the presence of miRNAs in single CTCs is evolving and has the potential to identify miRNAs that are essential for the maintenance of EMT and metastatic progression, which could be potential targets for treatment. The development of analyses of the epigenome that can be conducted on liquid biopsy samples is critical due to their non-invasive accessibility. We expect that single-cell epigenomics will enable us to map the changes in epigenetic marks throughout cancer evolution and identify the critical and highly dynamic epigenetic marks, paving the way for personalised therapies.

### Single-CTC transcriptomics

Single-cell RNA sequencing (scRNA-seq) provides high-resolution information on cellular differences that bulk RNA-seq does not reveal. Several methods have been developed for whole-transcriptome analysis (WTA), such as Smart-seq [172], Smart-seq2, Quartz-seq [173], CEL-seq [174] and STRT-seq [175]. The Smart-seq technologies utilise a unique reverse transcriptase that anchors to both cDNA ends, thereby increasing the number of full-length RNAs sequenced; this raises the efficiency of detection of alternatively spliced isoforms and genetic variants [176]. CEL-seq and STRT-seq target the 5’ ends and the 3’ ends of the mRNA, respectively, and are not suitable for detecting variants in the coding region or alternatively spliced transcripts [174, 177]. Each of these methods suffer from certain biases, and it is rare to achieve a full-length sequence from a single cell, and low-abundance transcripts are usually not detected [178].

Despite these current limitations, single-cell analysis at the transcriptomic level provides a novel tool for the identification of cell phenotypes and biomarkers. For instance, a combination of flow cytometry and high-density scRNA-seq identified molecular changes in CTCs from hepatocellular carcinoma (HCC) patients and oncogenic drivers such as IGF2 [179, 180]. This integrated approach could provide a novel tool for biomarker development in various cancers. scRNA-seq analysis of EMT phenotypes within CTCs across different vascular compartments revealed that CTCs predominantly have an epithelial phenotype at release but show EMT activation via Smad2 and β-catenin signaling during hematogenous transit [180]. A comprehensive scRNA-seq analysis of CTCs from mouse and human pancreatic ductal adenocarcinoma revealed enrichment of *SPARC*, which encodes an extracellular matrix-derived protein that enhances migration and invasion properties of cancer cells [25]. Through scRNA-seq on CTCs from 13 patients with drug-resistant prostate cancer, a novel drug resistance mechanism involving non-canonical Wnt signaling was identified [181]. Another discovery made using scRNA-seq is that high plakoglobin levels are associated with CTC cluster formation and can contribute to the metastatic spread of breast cancer [16]. These findings could aid in the customisation of existing therapeutic interventions. The recent development of Hydro-seq, a scalable hydrodynamic scRNA-seq barcoding technique, shows promising efficiency for high throughput whole-transcriptome CTC analysis, which will allow analyses of higher numbers of CTCs more efficiently and will likely increase the discovery of rare clones that are critical for cancer progression and metastasis [182].

### Single-CTC proteomics

Single-cell proteomic techniques are indispensable tools for exploring cellular mechanisms and processes. Proteins carry out functions like signal transduction and regulation of transcription and cytokine/chemokine secretion and mediate cell migration and invasion. Moreover, despite having the same genomic sequences, CTC subpopulations can differ in function and phenotype; hence, proteomics research holds great promise for the characterisation of the functions of CTCs. Current proteome profiling technologies can be broadly divided into cytometry-based and microfluidics-based platforms.

Microfluidics-based immunofluorescence techniques combine spatial and spectral encoding approaches and can detect up to 42 secreted proteins from a single cell [183]. Microfluidic western blotting integrates electrophoretic separations and multiple-cycle immunoassays, thereby allowing protein measurement in single rare cells such as CTCs. Sinkala and colleagues utilised single-cell resolution western blot [184] to evaluate a panel of eight surface and intracellular proteins in single CTCs isolated from patients with primary ER^+^ breast cancer and identified differences in expression patterns [185]. Single-cell western blot data are archivable and can be expanded to characterise subpopulations like CTCs undergoing EMT/immune escape and stem-like CTCs. Wang et al. developed a microchip-assisted single-cell proteomic method based on antibody and cellular DNA barcoding strategy for profiling CTC surface proteins [186]. However, these immunoassay-based approaches have shortcomings, including detecting a limited number of proteins and the need for antibodies [187].

Use of cytometry by time-of-flight (CyTOF) mass spectrometry for protein analysis has rapidly moved from the research laboratory to the clinical settings. CyTOF methodologies allow for the highly multiplexed and quantitative measurement of 35 or more proteins in a single cell [188]. Imaging mass cytometry employs mass spectroscopy-based analysis of tissue sections to yield information on the spatial expression of tens of proteins simultaneously [189]^,^. IMC allows for simultaneous detection of multiple markers in a single cell coupled with greater protein quantification and co-localisation information compared to other methods [190]. Gerdtsson et al. combined high-definition single-cell analysis (HD-SCA) and imaging mass cytometry to evaluate subcellular localisation of markers of prostate cancer in a clonally related population of CTCs and disseminated tumour cells [176]. HD-SCA enables simultaneous classification and monitoring of cancer [191] [192].

Proteomics studies are far more challenging than nucleic acid analyses mainly due to the complexity of the proteome and the need for highly specific probes and amplification tools. Moreover, highly sensitive detection methods are urgently needed to allow the study of low-abundance proteins and post-translational modifications. The use of proteomics to study rare CTCs using panels that include established therapeutic markers will help discover novel markers and mechanisms behind CTC immune escape. Targeted single-CTC proteomics has the potential to crucial insights into the process of metastatic progression [193].

### Personalised precision medicine targeting CTCs

Liquid biopsies to analyse cell-free DNA, exosomes and CTCs present in the blood can provide information on the tumour in a far less invasive way than a tumour biopsy. Currently, the enumeration of CTCs in a blood sample is used to guide treatment decisions. Although many cancer patients have very low or undetectable levels of CTCs, the presence of CTCs at the time of cancer diagnosis or recurrence is correlated with prognosis, and sequential liquid biopsy collection is used to evaluate disease progression. Several studies determined that between 60 and 80% of breast early breast cancer patients have detectable CTCs [194, 195]. The risk of recurrence increases and overall survival is reduced when CTCs are present in the blood sample in these patients. The recurrence risk correlates with the number of CTCs detected: The more CTCs, the higher the risk of recurrence [194, 195]. However, although at higher risk when CTCs were present, over 88% of women with CTCs had no cancer recurrence after 3 years of follow-up and over 87% had no metastatic progression [195]. Patients with CTCs are usually treated with chemotherapy; many of these patients are overtreated and may unnecessarily suffer chemotherapy’s side effects and complications. The other side of the coin is that many patients have very low or undetectable levels of CTCs but progress and develop metastatic disease [195]. This stresses the point that a higher resolution characterisation of CTCs is necessary to establish a treatment plan for patients with CTCs.

Clinically useful single-cell ‘omics approaches that can reveal prognostic phenotypic information about CTCs are needed. Epithelial markers, such as cytokeratin and EpCAM, are often used to detect CTCs [196–198]. Currently, the only FDA-approved test for CTC detection is CellSearch by Janssen Diagnostics based on the selection of EpCAM^+^ CTCs [81]. CTCs that have progressed toward the mesenchymal state have low expression of these epithelial markers (Fig. 1), and these dangerous CTCs potentially go undetected with the consequence that a patient does not receive the appropriate treatment needed to prevent recurrence and progression. Better assays must be developed that detected multiple biomarkers that are indicative of whether CTCs will cause cancer progression and metastasis. Single-cell multi-omics analysis together with long-term follow-up data will be able to identify biomarkers associated with progression.

All single-cell technologies give molecular insight into the makeup of CTCs, which we can correlate with clinical predictors and outcomes to identify CTC features that are detrimental for patients with solid cancers. Targeting these CTCs is challenging, not only because of our limited knowledge of which CTCs cause metastasis but also due to their heterogeneity. In-depth analyses of CTCs might provide insight into the cells that drive metastatic progression since the subclones that can become CTCs are subclones within the tumour. The true drivers of cancer progression might go undetected by performing multi-omics on the tumour bulk because these are rare clones with stem cell properties. The fitness to metastasize is represented in the CTCs, which can be observed in early disease before the existence of metastases. Likewise, drug resistance predictions could be determined based on the molecular makeup of CTCs. Over 90% of patients with cancer die of their metastases [199]. CTCs are the cells that cause metastasis and recurrences and thus should be eliminated to prevent metastatic progression and increase survival. CTC profiling should become an added tool together with current tools and approaches for an oncologist to determine patient diagnosis and prognosis. Like tumours, CTCs can have a very different molecular makeup between patients; hence CTC characterisation and application of personalised precision medicine go hand in hand. Until the true identity of CTCs responsible for cancer progression is identified, integration as part of the routine clinical analysis will be impossible. For CTC profiling to become an integrated part of routine clinical practice, a small biomarker set identifying and characterising CTCs is needed.

Targeting alterations in CTCs identified from the tumour with established compounds is an attractive approach to treat cancer. For instance, *HER2* is frequently amplified and overexpressed in breast cancer, and the detection of changes in *HER2* gene expression in CTCs might contribute to the effective stratification of breast cancer patients and personalised treatment strategies targeting HER2. Mutations in the *ESR1*, which encodes an estrogen receptor, can have large consequences on the efficacy of hormone therapy [200, 201]. These mutations can be detected in tumours, and two recent studies were able to identify activating mutations in *ESR1* in CTCs [202, 203]. CTCs isolated from colorectal cancer (CRC) patients harbored *KRAS* mutations, which are a strong predictor for resistance to EGFR inhibitors [204]. These studies demonstrate that applying current knowledge from tumours to CTCs can be efficacious when sensitive detection methods are used.

## Conclusion

Isolation and analysis of CTCs from liquid biopsies can offer non-invasive diagnostic and therapeutic information in a wide variety of cancers. Emerging research suggests the existence of different CTC subpopulations with differing phenotypic compositions and metastatic capabilities in individual patients. This underscores the importance of targeted studies to identify the phenotypes and genotypes of CTCs with specific emphasis on characterising their metastatic potential. Although the detection of CTCs remains a challenge due to low numbers of CTCs in the blood, recent advancements in isolation methods and analysis technologies suggest that CTC phenotyping will soon be used clinically.

Characterisation of single CTCs via the integration of genomic, epigenomic, transcriptomic and proteomic approaches has shed much light on intra-tumour heterogeneity and therapeutic resistance mechanisms (Table 1) [141, 205–209]. Single-cell multi-omics approaches have the potential to map the evolution of CTCs and thereby build an atlas of tumour evolution that includes a plethora of therapeutically viable targets. Profiling of CTC-derived biomarkers holds greater clinical significance than the current diagnostic and prognostic tools alone and their circulating characteristic is a considerable advantage. With the current pace of CTC research and the potential for the discovery of biomarkers for CTCs likely to cause cancer progression, liquid biopsies have the potential to become a routine for screening and monitoring cancer patients, paving the way toward more personalised therapies.

**Table 1:** Multi-omics strategies for analyses of single CTCs.

### Reporting summary

Further information on research design is available in the Nature Research Reporting Summary linked to this article.

## Supplementary information

Reporting Summary
